# Prevalence and factors associated with COVID-19 vaccine acceptance among the general population in Asadabad, Iran: a cross-sectional study

**DOI:** 10.1186/s41182-022-00453-0

**Published:** 2022-08-30

**Authors:** Ahmed Najeeb Albatineh, Pegah Dalvand, Marzieh Aslani, Serdar Saritas, Vajiheh Baghi, Reza Ghanei Gheshlagh

**Affiliations:** 1 Department of Community Medicine and Behavioural Sciences, Faculty of Medicine, Kuwait University, Kuwait City, Kuwait; 2 Department of Mathematics, Shahrood University of Technology, Shahrood, Iran; 3 Department of Nursing, Asadabad School of Medical Sciences, Asadabad, Iran; 4 Department of Surgical Nursing, Faculty of Nursing, Inonu University, Campus 44280, Malatya, Turkey; 5 Be’sat Hospital, Kurdistan University of Medical Sciences, Sanandaj, Iran; 6 Social Determinants of Health Research Center, Research Institute for Health Development, Kurdistan University of Medical Sciences, Sanandaj, Iran

**Keywords:** COVID-19, COVID-19 vaccine acceptance, Vaccination, Iran

## Abstract

**Background:**

Vaccination can be an essential protective measure against Coronavirus disease 2019 (COVID-19) if well received by the public. Various factors affect the acceptance or refusal of vaccines. Several waves of COVID-19 caused much death in Iran. This study aimed to evaluate the acceptance of the COVID-19 vaccine in the general population of Asadabad in 2021.

**Methods:**

In this cross-sectional study, 650 people from the general population of Asadabad with a mean age of 34.6 (SD = 15.1) years were selected and included. In addition to socio-economic and demographic data, data were collected using the COVID-19 fear scale. Univariate and multiple logistic regression models were used to investigate the relationship between the tendency to get the COVID-19 vaccine (the dependent variable) and other variables.

**Results:**

About 42.3% of participants were reluctant to receive the available COVID-19 vaccines. After adjusting for several covariates, there was a significant relationship between willingness to get vaccinated and family history of COVID-19 infection (AOR = 1.86, 95% CI 1.06–3.27, *p* = 0.032), trust in healthcare workers (AOR = 2.07, 95% CI 1.13–3.79, *p* = 0.019), trust in existing vaccines (AOR = 3.94, 95% CI 2.15–7.23, *p* < 0.001), encouraging family members to get vaccinated (AOR = 7.6, 95% CI 4.12–14.01, *p* < 0.0001). Also, people infected with COVID-19 are less likely to accept vaccination (AOR = 0.55, 95% CI 0.33–0.93, *p* = 0.025). Also, a unit increase in the score of fear of getting the COVID-19 virus increased the odds of getting the COVID-19 vaccine by 6% (AOR = 1.06, 95% CI 1.02–1.10, *p* = 0.002).

**Conclusion:**

The culture and context of different societies can affect the acceptance or refusal of the COVID-19 vaccine. Based on these characteristics and providing extensive education to the people, the health authorities in each community should build trust and better communicate all health information to clear any fear and remove all obstacles to increase willingness to get COVID-19 vaccination.

## Introduction

The rapid spread of Coronavirus disease 2019 (COVID-19) has led to a panic-stricken pandemic worldwide [1]. To control the spread of COVID-19, governments took precautionary measures such as social distancing, quarantine of suspected and confirmed cases, travel restrictions, strict lockdowns, mandatory mask use, and hygiene [2]. Although these measures were preventive and valuable, they changed people's lifestyles and affected their physical, mental, and social well-being [3]. Finally, mass vaccination was proposed as the most effective approach to control the spread and severity of the COVID-19 pandemic [4].

Vaccination is one of the most outstanding public health achievements that has reduced mortality from many infectious diseases and led to the elimination of poliomyelitis in the United States and the eradication of smallpox worldwide. If vaccination programs have a high uptake level, the prevalence and incidence of vaccine-preventable diseases (VPD) will undoubtedly be reduced. High vaccination coverage directly protects vaccinated individuals and indirectly exerts protective effects by slowing the transmission of VPD from the general population (herd safety) [5]. Most experts believe that without widespread acceptance of vaccination, the COVID-19 pandemic cannot be contained [6], and vaccination can quickly and efficiently reduce the epidemic’s burden [7, 8]. By June 12, 2021, more than 2.36 billion vaccine doses have been injected worldwide [9]. Because some people were skeptical about receiving the vaccine and some refused, there was a considerable gap between vaccination programs in different countries [10]. Vaccination hesitancy refers to any delay in accepting or refusing a vaccine despite the availability of vaccination services, which is a growing problem [11]. Doubts about vaccination are not a new phenomenon. For example, the poliomyelitis vaccination program in Pakistan faced challenges like poor vaccine quality, an active virus in the vaccine, and the ban on this vaccine from a religious point of view [12]. Another example of vaccine hesitancy occurred during the 2009 influenza A epidemic, in which officials in many parts of the Americas struggled to persuade pregnant women to get the vaccine [13]. Reluctance to getting the measles vaccine in parts of Europe, the Human papillomavirus (HPV) vaccine in Japan and India [14], polio vaccine in parts of Nigeria [15], and Pakistan [16] are recent examples of vaccine hesitancy.

There were six waves of COVID-19 in Iran at the following times: March to June 2020 (first wave), July to September 2020 (second wave), October to December 2020 (third wave), April to June 2021(fourth wave), July to September 2021(fifth wave), and March 2022 (sixth wave). The seventh wave is already starting in Iran. By August 2022, 58.1 million people in Iran have been injected with at least one dose of the COVID-19 vaccine, and 69.1% have been fully vaccinated (received all doses) [17].

There are similar ideas about the COVID-19 vaccine. Misinformation about the COVID-19 pandemic on social media, such as linking the virus to 5G cell phone networks, the premature death of vaccinated people, or the pandemic is a terrorist weapon, made many people reluctant to get vaccinated [18]. Given that the decision to receive a vaccine is a complex interaction between different social, cultural, political, and personal factors, it is difficult to have a clear picture of possible attitudes about vaccination in the general population. This study investigated the prevalence of acceptance and refusal of COVID-19 vaccination and its related factors in the general population of Asadabad (one of the western cities of Iran).

## Methods

### Sample and setting

This cross-sectional study was conducted in November and December 2021 in Asadabad. The study sample consisted of 650 people from the general population selected by convenience sampling method from public places. Sampling was done in person. Inclusion criteria were: the ability to read and write, not receiving the COVID-19 vaccine until the time of the study, and willingness to participate. Incomplete questionnaires were excluded from the analysis.

### Instrument

We used a form based on a literature review and consultation with several healthcare professionals to collect demographic information. The information contained in this form included: age, gender, marital status, education, underlying disease, smoking, history of infection of a person or a family member with COVID-19, death of a family member due to COVID-19, the existence of a family member as a health specialist, the impact of COVID-19 on quality of life, trust in the healthcare workers, trust in existing vaccines, advise family members to get the COVID-19 vaccine, and fear of COVID-19. To measure people's fear of COVID-19, we used the Fear of COVID-19 Scale (FCV-19S), which included seven questions with a 5-point Likert answer (from strongly agree to strongly disagree). The scores range from zero to 35, so a higher score indicates much fear. This scale is available in both Persian and English versions, and its psychometric properties have been confirmed and reported [19].

### Data analysis

We reported the qualitative and quantitative variables as number and percentage, mean and standard deviation, respectively. Univariate and multiple logistic regression models were used to investigate the relationship between the tendency to get the COVID-19 vaccine and other variables. Firstly, in the univariate model, the relationship between the tendency to get the COVID-19 vaccine with each of the independent variables of the model was investigated.

We entered all variables with a *p*-value ≤ 0.25 into multiple logistic regression analysis to control the effect of potential confounders. This method ensures that no covariate has been ignored due to an insignificant p-value in the univariate analysis. Finally, a stepwise logistic regression model was run to identify the most parsimonious model that included all covariates that could predict the acceptability of the COVID-19 vaccine. We used the backward method using the likelihood ratio approach, where the probability of entry was set at 0.40 and the exit at 0.10, so no significant covariates were omitted. All tests were two-tailed, and the significance level was 5%.

### Ethical considerations

The objectives of the study were explained to all participants and their consent to participate was obtained. All questionnaires were distributed anonymously and participants were assured that all their information would remain confidential. The Ethics Committee of Asadabad University of Medical Sciences (No. IR.ASAUMS.REC.1401.007) has approved this study. All procedures were performed in this study following the 2013 Declaration of Helsinki. Consent was obtained from all participants.

## Results

The samples included 326 females and 324 males with a mean age of 34.6 (DS = 15.1), ranging in age from 10 to 91 years. Most of the participants (51.4%) were married, had university education (43.7%), were non-smokers (76.6%), and had no underlying diseases (76%). More than half of the participants had a history of COVID-19 infection (51.2%), and 69.8% of the participants stated that one of their family members had a history of COVID-19 infection. Also, 15.1% of the participants had lost a loved one due to COVID-19. More than a third (38.3%) also reported that a family member had been hospitalized due to a COVID-19 infection. On the other hand, 49.5% and 30.7% of the participants believed that the COVID-19 pandemic badly impacted their quality of life and income. More than a quarter of the participants did not trust health professionals, and about 40% did not trust the available vaccines. More than half of the participants (57.7%) would have liked to be vaccinated if there was a trusted vaccine. The mean score of COVID-19 fear among participants was 20.34 ± 6.98. Further details are provided in Table 1.

**Table 1:** The demographic profile of the study participants (*N* = 650)

Variables	*N*	%
Gender		
Male	324	49.8
Female	326	50.2
Marital status		
Single	316	48.6
Married	334	51.4
Literacy		
Primary and secondary	156	24
High school	210	32.3
Academic	284	43.7
Underlying disease		
Yes	156	24
No	494	76
Smoking		
Smoker	100	15.4
Ex-smoker	52	8
Non-smoker	498	76.6
History of COVID-19 infection		
Yes	333	51.2
No	317	48.8
Existence of a family member as a health specialist		
Yes	200	30.7
No	450	69.3
Family history of COVID-19		
Yes	453	69.8
No	197	30.2
Death of a family member due to COVID-19		
Yes	98	15.1
No	552	84.9
Family history of hospitalization due to COVID-19		
Yes	249	38.3
No	401	61.7
History of influenza vaccine injection		
Yes	245	37.7
No	405	62.3
The effect of COVID-19 on quality of life		
Low	75	11.5
Medium	253	39
High	322	49.5
The effect of COVID-19 on your income		
Low	200	36.2
Medium	215	33.1
High	235	30.7
Trust in healthcare workers		
Yes	482	74.2
No	168	25.8
Trust in existing vaccines		
Yes	388	59.7
No	262	40.3
Advise family members to get the COVID-19 vaccine		
Yes	413	63.7
No	237	36.3

According to the results reported in Table 2, in the univariate model, all the variables except a history of COVID-19 infection (*p*-value = 0.888), the existence of a family member as a health specialist (*p*-value = 0.388), family history of hospitalization due to COVID-19 (*p*-value = 0.787), and effect of COVID-19 on your income (*p*-value = 0.733) were entered into multiple logistic regression model for analysis. Accordingly, after adjusting for all other covariates included in the analysis, people who lost a family member to COVID-19 were 56% less likely to accept vaccination than others who did not lose a family member due to COVID-19. Also, the odds of vaccination acceptance in people who have trust in healthcare workers and trust in the vaccine were 2.07 times that for those who have no trust (95% CI 1.13–3.79, *p* = 0.019) and 3.94 (95% CI 2.15–7.23, *p* < 0.001), respectively. The odds of vaccine acceptance in people who were encouraged by their families to get the vaccine were 7.6 times higher than in others (95% CI 4.12–14.01, *p* < 0.0001). Also, for a unit increase in fear of COVID-19 score, there was an increase in the odds of vaccine acceptance by 6% (OR = 1.06, 95% CI 1.02–1.10, *p* = 0.002) (Table 2). Finally, the multiple logistic regression model was significant according to the omnibus test (χ^2^ = 360.75, dof = 16, *p* < 0.001), and according to Hosmer–Lemeshow test the model fits the data well (χ^2^ = 7.23, dof = 8, *p* = 0.512) with a ROC value of 0.891 (95% CI 0.863–0.918) indicating excellent discrimination of the study participants.

**Table 2:** Univariable and multivariable logistic regression modeling for the association between COVID-19 vaccine acceptance and several covariates (N = 650)

Variable	Univariable logistic regression		Multivariable logistic regression	
	OR (95% CI)	p-value	OR (95% CI)	*p*-value
Age	0.98 (0.97–0.99)	0.001	1.00 (0.97–1.01)	0.645
Gender				
Female	1.65 (1.21–2.27)	0.002	1.18 (0.72–1.93)	0.499
Male	Ref		Ref	
Marital status				
Single	1.34 (0.98–1.84)	0.064	0.92 (0.54–1.56)	0.750
Married	Ref		Ref	
Education				
High school	1.15 (0.76–1.74)	0.509	1.12 (0.58–2.13)	0.738
Academic	1.64 (1.11–2.44)	0.014	1.27 (0.69–2.33)	0.437
Elementary	Ref		Ref	
Underlying disease				
Yes	0.70 (0.49–1.01)	0.056	0.89 (0.50–1.60)	0.704
No	Ref		Ref	
Smoking status				
Current Smoker	0.42 (0.27–0.66)	< 0.0001	0.83 (0.43–1.61)	0.578
Previous smoker	0.40 (0.22–0.71)	0.002	0.67 (0.26–1.70)	0.395
Non-smoker	Ref		Ref	
History of COVID-19 infection				
Yes	1.02 (0.75–1.40)	0.888	–	–
No	Ref		Ref	
Existence of a family member as a health specialist				
Yes	1.16 (0.83–1.63)	0.388	–	–
No	Ref		Ref	
Family history of hospitalization due to COVID-19				
Yes	0.97 (0.69–1.32)	0.787	–	–
No	Ref		Ref	
Death of a family member due to COVID-19				
Yes	0.66 (0.43–1.02)	0.059	0.44 (0.23–0.82)	**0.010**
No	Ref		Ref	
History of influenza vaccine injection				
Yes	0.80 (0.58–1.10)	0.172	–	–
No	Ref		Ref	
The effect of COVID-19 on quality of life				
Very	1.42 (0.86–2.35)	0.170	–	–
Middle	1.51 (0.90–2.53)	0.119	–	–
Low	Ref		Ref	
The effect of COVID-19 on your income				
Very	0.91 (0.62–1.34)	0.649	–	–
Middle	1.06 (0.72–1.57)	0.756	–	–
Low	Ref		Ref	
Trust in healthcare workers				
Yes	2.31 (1.88–2.74)	< 0.0001	2.07 (1.13–3.79)	**0.019**
No	Ref		Ref	
Trust in existing vaccines				
Yes	3.10 (2.66–3.47)	< 0.0001	3.94 (2.15–7.23)	**< 0.0001**
No	Ref		Ref	
Advise family members to get the COVID-19 vaccine				
Yes	3.37 (2.92–3.81)	< 0.0001	7.60 (4.12–14.01)	** < 0.0001**
No	Ref		Ref	
Fear of COVID-19	1.10 (1.05–1.10)	< 0.0001	1.06 (1.02–1.10)	**0.002**

The “–” symbol indicates that the covariate was not included in the multiple logistic regression model because its univariate *p* value was > 0.25

The results of the stepwise logistic regression method using backward elimination with a likelihood ratio approach are presented in Table 3. Results showed that people with a history of COVID-19 infection have lower odds of vaccine acceptability (AOR = 0.55, 95% CI 0.39–0.93, *p* = 0.025) and those with a family history of COVID-19 infection have higher odds of vaccine acceptability (AOR = 1.86, 95% CI 1.06–3.27, *p* = 0.032). Additionally, people with families who died due to COVID-19 have lower odds of vaccine acceptability (AOR = 0.44, 95% CI 0.23–0.84, *p* = 0.013), while those who trust healthcare workers have higher odds of vaccine acceptability (AOR = 2.04, 95% CI 1.1–3.75, *p* = 0.024). Furthermore, people who have trust in the available vaccine and have advice from a family member to get the vaccine to have higher odds of vaccine acceptability with (AOR = 3.55, 95% CI 1.93–6.52, *p* < 0.001) and (AOR = 9.56, 95% CI 5.20–17.57, *p* < 0.001), respectively. Finally, for a one-unit increase in fear of COVID-19, there was 6% increase in the odds of vaccine acceptability (AOR = 1.06, 95% CI 1.02–1.09, *p* = 0.001).

**Table 3:** Results from the stepwise logistic regression modeling by backward elimination method with likelihood ratio method using all covariates (*N* = 650)

Covariate	Adjusted OR (95% CI)	*p*-value
History of COVID-19 infection		
Yes	0.55 (0.33–0.93)	0.025
No	Ref	
Family history of hospitalization due to COVID-19		
Yes	1.86 (1.06–3.27)	0.032
No	Ref	
Death of a family member due to COVID-19		
Yes	0.44 (0.23–0.84)	0.013
No	Ref	
Trust in existing vaccines		
Yes	3.55 (1.93–6.52)	< 0.001
No	Ref	
Trust in healthcare workers		
Yes	2.04 (1.10–3.77)	0.024
No	Ref	
Advise family members to get the COVID-19 vaccine		
Yes	9.56 (5.12–17.57)	< 0.001
No	Ref	
Fear of COVID-19	1.06 (1.02–1.09)	0.001

## Discussion

This study aimed to evaluate the acceptance of COVID-19 vaccination in the general population of Asadabad. Results showed that the death of a family member due to COVID-19, trust in healthcare workers, trust in existing vaccines, advice to family members to get the COVID-19 vaccine, and fear of COVID-19 were significantly associated with vaccine acceptance.

Acceptance of vaccination is a behavioral consequence of a complex decision-making process that various factors can potentially influence. In the present study, about 42.3% of participants were reluctant to receive the COVID-19 vaccine. All attitudes towards vaccination can be seen, from active demand to complete refusal. Vaccine hesitators are generally a heterogeneous group in the middle of the continuum who may refuse some vaccines and accept others [20].

These people are more skeptical about newer vaccines [21, 22]. The low acceptance of vaccines seems to be that people are skeptical of new vaccines as a new technology because no previous past experience or success with such an approach has been reported. Another reason could be the high speed of production and registration of vaccines (in less than a year), which can reduce the acceptance of these vaccines [23].

People who trusted healthcare workers were twice as likely to be vaccinated. A study in Saudi Arabia showed that 64.7% of people were willing to be vaccinated, and people who trusted the health system were three times more likely to be vaccinated [24]. In the study by Tran et al. (2021), the odds of receiving the vaccine in people who trusted the medical staff were 2.7 times higher than in others [25]. People who were afraid of COVID-19 were also more likely to be vaccinated. In the study of Ahmad et al. in Pakistan, 62% of the participants were willing to receive the COVID-19 vaccine. There was a significant relationship between acceptance of the COVID-19 vaccine and fear of COVID-19 [26]. People who trusted the vaccine were nearly four times more likely to get the COVID-19 vaccine. This finding makes sense because trust in the vaccine leads to vaccine acceptance. Also, the chance of getting the vaccine in people who encouraged their families to get the vaccine was 7.6 times higher than in others. A person who trusts the available vaccines will encourage his family to get the vaccine, and he will get the vaccine according to his family members. Results indicated that people with a history of COVID-19 infection have lower odds of vaccine acceptability as they may think that they already have immunity due to their infection. Those with a family history of COVID-19 infection have higher odds of vaccine acceptability because they might have seen the suffering of their family member due to COVID-19 infection. One of the strange findings of this study was the low odds of vaccine acceptance in people who had lost a family member to COVID-19. The reason for this finding may be that the guilt is lost due to the loss of a loved one, and with this immature mechanism, people try to reduce their guilt through self-punishment. Also, due to the severity of the discomfort, the person may not have the motivation to continue living and would like to experience this disease and its consequences.

A new vaccine should be accepted by at least 70% of the population to create herd immunity, which sometimes reaches 85% depending on the type of country and the infection rate [27, 28]. Large-scale vaccine refusal can be a threat to herd immunity. On the other hand, large-scale acceptance of local vaccine refusal can adversely affect community safety because non-vaccinators can inappropriately increase the vaccination coverage required to achieve herd safety [18].

## Strength and limitations

One of the strengths of this study is the comprehensiveness and accuracy in completing the questionnaires. Also, the trained samplers, in addition to delivering the questionnaires to the people, guided them carefully and answered their possible questions. One of the limitations of this study was the periods of intermittent access to the vaccine. Sometimes the vaccine was scarce, and sometimes it was abundant. Also, some participants stated that the vaccine they were injected with was fake and had to go to health centers for re-vaccination. Another limitation was the society's culture of mistrust, as some participants witnessed the death of their loved ones. At the same time, they had already been vaccinated, thus attributing this situation to the vaccines being undesirable. According to the experience of different waves of the COVID-19 pandemic, people were no longer as afraid of this disease as before; in other words, they had lost their fear with time. Therefore, the time of conducting the study can affect our results, which was beyond the control of the researchers.

## Conclusion

This study showed that less than half of the participants were willing to be vaccinated, and this reluctance is influenced by society's experiences, culture, and context. Therefore, the health officials of each society must take measures to remove the obstacles to vaccine injection based on these characteristics. Also, it seems necessary to provide public education to encourage people to get vaccinated and build confidence in people about the available vaccines.

## Data Availability

The datasets used and/or analyzed during the current study are available from the corresponding author on reasonable request.

## Abbreviations

COVID-19: Coronavirus disease 2019
VPD: Vaccine-preventable diseases
